# The prevalence, presentation and outcome of colistin susceptible-only *Acinetobacter Baumannii*-associated pneumonia in intensive care unit: a multicenter observational study

**DOI:** 10.1038/s41598-022-26009-0

**Published:** 2023-01-04

**Authors:** Sheng-Huei Wang, Kuang-Yao Yang, Chau-Chyun Sheu, Yu-Chao Lin, Ming-Cheng Chan, Jia-Yih Feng, Chia-Min Chen, Chih-Yu Chen, Zhe-Rong Zheng, Yu-Ching Chou, Chung-Kan Peng

**Affiliations:** 1 Division of Pulmonary and Critical Care Medicine, Department of Internal Medicine, Tri-Service General Hospital, National Defense Medical Center, No. 325, Section 2, Cheng-Gong Rd, Neihu 114, Taipei, Taiwan; 2 Graduate Institute of Medical Sciences, National Defense Medical Center, Taipei, Taiwan; 3 Department of Chest Medicine, Taipei Veterans General Hospital, Taipei, Taiwan; 4 Institute of Emergency and Critical Care Medicine, School of Medicine, National Yang Ming Chiao Tung University, Taipei, Taiwan; 5 Cancer Progression Research Center, National Yang Ming Chiao Tung University, Taipei, Taiwan; 6 Division of Pulmonary and Critical Care Medicine, Department of Internal Medicine, Kaohsiung Medical University Hospital, Kaohsiung Medical University, Kaohsiung, Taiwan; 7 Department of Internal Medicine, School of Medicine, College of Medicine, Kaohsiung Medical University, Kaohsiung, Taiwan; 8 Division of Pulmonary and Critical Care Medicine, Department of Internal Medicine, China Medical University Hospital, Taichung, Taiwan; 9 School of Medicine, China Medical University, Taichung, Taiwan; 10 Department of Critical Care Medicine, Taichung Veterans General Hospital, Taichung, Taiwan; 11 School of Post Baccalaureate Medicine, National Chung Hsing University, Taichung, Taiwan; 12 School of Medicine, National Yang Ming Chiao Tung University, Taipei, Taiwan; 13 Division of Pulmonary Medicine, Department of Internal Medicine, Chung Shan Medical University Hospital, Taichung, Taiwan; 14 Division of Chest Medicine, Department of Internal Medicine, Taichung Veterans General Hospital, Taichung, Taiwan; 15 School of Public Health, National Defense Medical Center, Taipei, Taiwan

**Keywords:** Infectious diseases, Respiratory tract diseases

## Abstract

Hospital-acquired pneumonia (HAP) and ventilator-associated pneumonia (VAP) caused by carbapenem-resistant *Acinetobacter baumannii* (CRAB) are both associated with significant morbidity and mortality in daily clinical practice, as well as in a critical care setting. It is unclear whether colistin susceptible-only *Acinetobacter baumannii* (CSO AB) is a unique phenotype separate from or a subset of CRAB-associated pneumonia. The aim of this study is to investigate the prevalence of CSO AB pneumonia and compare the presentation and outcome between CSO AB and CRAB-associated pneumonia in critically ill patients. This multicenter retrospective cohort study initially recruited 955 patients with CR-GNB pneumonia. After exclusion, 575 patients left who were ICU-admitted and had CRAB nosocomial pneumonia remained. Among them, 79 patients had CSO AB pneumonia, classified as the CSO AB group. The other 496 patients were classified as the CRAB group. We compared demographic characteristics, disease severity, and treatment outcomes between the two groups. The prevalence of CSO AB among all cases of CRAB pneumonia was 13.74% (79/575). The CSO AB and CRAB groups had similar demographic characteristics and disease severities at initial presentation. The in-hospital mortality rate was 45.6% and 46.4% for CSO AB and CRAB groups, respectively (p = 0.991). The CSO AB group had significantly better clinical outcomes at day 7 (65.8% vs 52.4%, p = 0.036) but longer length of ICU stay (27 days vs 19 days, p = 0.043) compared to the CRAB group. However, other treatment outcomes, including clinical outcomes at day 14 and 28, mortality, microbiological eradication, ventilator weaning, and newly onset dialysis, were similar. In conclusion, CSO AB accounted for 13.74% of all cases of CRAB pneumonia, and the clinical presentation and treatment outcomes of CSO AB and CRAB pneumonia were similar.

## Introduction

*Acinetobacter baumannii* (*A. baumannii*) can cause nosocomial infections involving multiple organ systems, including bloodstream, respiratory tract, urinary tract, skin and soft tissue, and central nervous system infections, among others. Those resulted in significant morbidity and mortality in healthcare institutions around the world^1^. Among these, *A. baumannii*-associated ventilator-associated pneumonia (VAP) and bacteremia were the cause of the high mortality rate that was approximately 40–60% within one year^2–5^. In addition to multifactorial virulence factors and tenacity for survival in different environments, versatile resistance mechanisms threatening the antibiotic therapy makes *A. baumannii* infection a critical and troublesome clinical entity^6^. The advert of carbapenem-resistant organisms such as carbapenem-resistant *Acinetobacter baumannii* (CRAB), carbapenem-resistant *Pseudomonas aeruginosa* and carbapenem-resistant *Enterobacterales* breaks the fortress of carbapenem, a reliable last-resort for antimicrobial therapy, and makes a global public-healthcare problem^7^. In 2016, the World Health Organization (WHO) announced the priority list of drug-resistant bacteria for research into and development of effective antibiotics; CRAB was indicated as one of the bacteria with the highest priority^8^.

The treatment for CRAB pneumonia is usually a regimen of single or combination antibiotics, including colistin, tigecycline, sulbactam, carbapenem, amikacin, minocycline, fosfomycin, etc.; promising agents include cefiderocol and eravacycline^9–11^. In CRAB pneumonia, colistin susceptible-only *Acinetobacter baumannii* (CSO AB) is a subgroup that has not been thoroughly studied. Although colistin is one of the mainstay treatments for CRAB pneumonia and specifically effective for CSO AB pneumonia, CSO AB pneumonia is a clinical entity worthy of discussion. If clinicians could know the epidemiology of CSO AB pneumonia and early detect the risk factors and initial presentations of CSO AB pneumonia in healthcare institutions, early prescription of colistin for critically ill patients before the availability of antimicrobial susceptibilities may lead to better clinical outcomes. In 2007, prior antimicrobial therapy for more than 10 days and a previous VAP episode were reported by Rios to be the independent risk factors of acquiring CSO AB-associated VAP, which clues clinicians into prescribing colistin for critically ill patients relatively early in the disease course^12^. However, nowadays the prevalence, presentation, and prognosis of CSO AB-associated nosocomial pneumonia in intensive care unit (ICU), which are needed for more effective treatment of resistant *A. baumannii* infection, are still unclear. For this information gap, we conducted a multicenter retrospective cohort study to investigate the ratio of CSO in CRAB pneumonia, and to compare the clinical manifestation and outcomes between CSO AB and CRAB pneumonia in patients admitted to the ICU.

## Methods

### Study population and data collection

**Figure 1:**
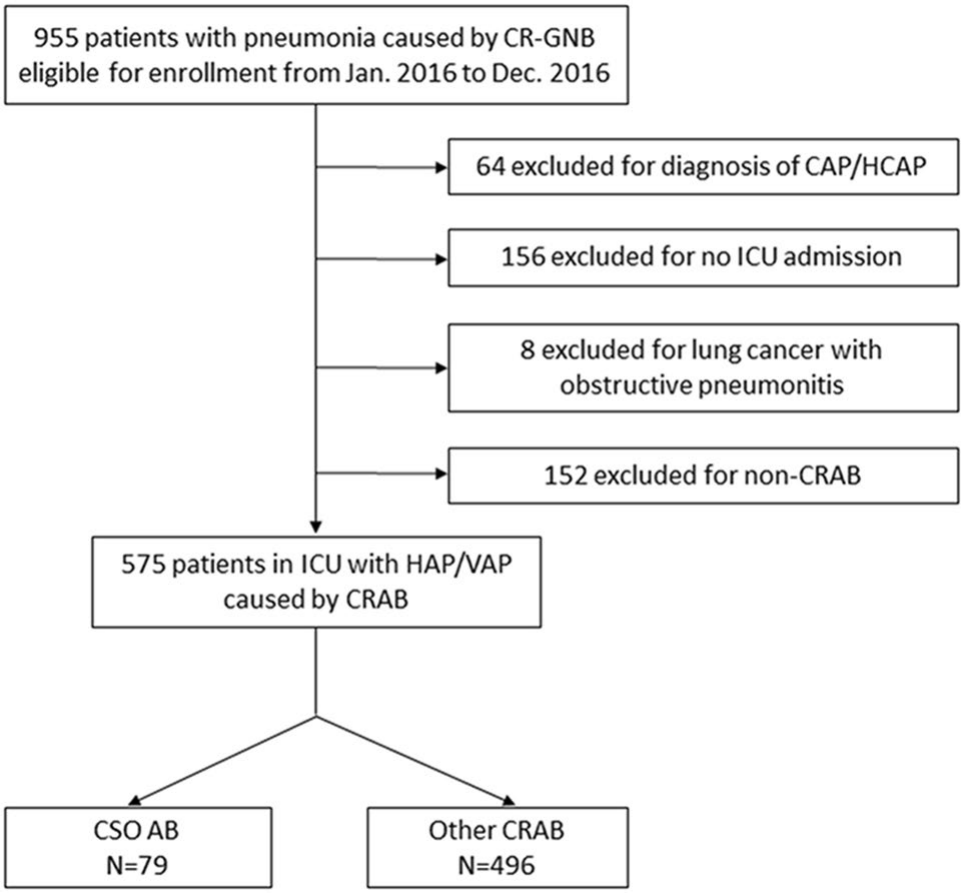
Flow diagram for patient inclusion and exclusion.

This is a retrospective study conducted in five medical centers in Taiwan^13,14^. We recruited ICU-admitted patients who were diagnosed with carbapenem-resistant Gram-negative bacterial (CR-GNB) associated pneumonia from Jan. 2016 to Dec. 2016. The study design has been mentioned in the article published by team members^15^, and we briefly described it in this method section. The CSO AB and CRAB pneumonia were derived from a CR-GNB pneumonia database. The flow diagram of this article for patient inclusion and exclusion is shown in Fig. 1. The inclusion criteria were ICU admission with a diagnosis of nosocomial pneumonia that developed more than 48 h after admission, and the culture of CR-GNB from respiratory specimens that was resistant to at least one kind of tested carbapenems. The exclusion criteria were age younger than 20 years, community-acquired pneumonia or healthcare-associated pneumonia, concomitant lung cancer with obstructive pneumonitis, and non-CRAB pathogens.

The baseline characteristics, disease severities and clinical outcomes were retrieved from the medical records of the five teaching hospitals as listed on the affiliation. The disease severities of recruited patients were assessed by the Sequential Organ Failure Assessment (SOFA) score on the day of ICU admission and pneumonia index date, and the Acute Physiology and Chronic Health Evaluation (APACHE) II score on the day of ICU admission. We also analyzed the parameters associated with the organ dysfunction, including septic shock, PaO2/FiO2 (P/F) ratio, use of mechanical ventilator, and renal replacement therapy, and the laboratory studies including plasma leukocyte, C-reactive protein, albumin and creatinine on the pneumonia index date. Septic shock was defined according to the International Sepsis Definitions Conference in 2001^16^.

### Diagnosis of nosocomial pneumonia

Pneumonia was diagnosed upon discovery of new or progressive infiltration, consolidation or patch opacity on chest x ray with at least two clinical observations, including fever (> 38 °C) or hypothermia (< 36 °C), cough, copious or purulent phlegm secretion, leukocytosis (plasma leukocyte > 10,000/mm^3^), leukopenia (plasma leukocyte < 4000/mm^3^), or percentage of band form leukocyte > 10%. In this study, we recruited patients with nosocomial pneumonia including hospital-acquired pneumonia which was defined as pneumonia that occurred more than 48 h after hospital admission, and VAP which was defined as pneumonia that developed more than 48 h after endotracheal intubation. We just recorded and collected the first episode of HAP or VAP caused by CRAB infection for subsequent analysis. Eligible specimens were collected from sputum (semi-quantitative method), tracheal aspirates (semi-quantitative method), or bronchoalveolar lavage fluid with a CR-GNB concentration > 10^4^ colony forming units per ml (CFU/mL). We defined the date of specimen collection as the pneumonia index date (pneumonia onset day).

### Microbiological tests and therapeutic regimens

The antibiotic susceptibility of the causative *A. baumannii* was determined based on the Clinical and Laboratory Standards Institute (CLSI) recommendations. Either the BD Phoenix™ system or the VITEK® 2 system was used for antimicrobial susceptibility tests in this study. The antibiotics tested for susceptibility included piperaci/tazoba, ampicillin/sulbactam, cefepime, ceftazidime, levofloxacin, ciprofloxacin, fosfomycin, meropenem, imipenem, doripenem, gentamicin, amikin, tigecycline and colistin. Antimicrobial susceptibility such as colistin, tigecycline, ampicillin/sulbactam, and amikacin for each CRAB was recorded for analysis. *A. baumannii* was defined as carbapenem-resistant (CRAB) when *A. baumannii* was resistant to at least one kind of tested carbapenems (meropenem, imipenem, doripenem); *A. baumannii* was defined as colistin susceptible-only (CSO) when *A. baumannii* was resistant to all tested antibiotics but only susceptible to colistin. We recorded the antibiotics commonly prescribed for CRAB treatment for baseline characteristic analysis, including carbapenem, colistin (intravenous or inhaled), tigecycline, sulbactam, and amikacin, that should be administered for at least 2 days within 7 days of the pneumonia index date; antibiotics that were administered less than 2 days were not recorded in this study.

### Outcomes evaluations

The mortality rate, favorable clinical outcome, and microbiological response at days 7, 14, and 28 were the primary outcomes in the present study. We followed up the patients to discharge and outpatient-department visit according to medical record (retrospectively). We classified the clinical response to treatment as either cure (discontinuation of antibiotic and resolution of symptoms), improvement (continuation of antibiotics treatment and partial resolution of symptoms), or failure (persisted symptoms or decease). The symptoms for the assessment of clinical response included fever or hypothermia, cough, copious or purulent phlegm secretion. The combination of cure and improvement were defined as favorable clinical outcome. We classified the microbiological response as either eradication (no yield of causative pathogens in two or more consecutive respiratory specimens), persistence (persistent yield of causative pathogens in respiratory specimens), recurrence (isolation of causative pathogens again within 14 days of eradication), or undetermined (no follow-up specimen or only one specimen without pathogen growth). We defined the microbiological eradication rate as the ratio between the cases with eradiation and the cases with eradiation, persistence, or recurrence (undetermined was not included).

The 28-day ventilator weaning, newly onset dialysis, length of ICU and hospital stay, and length of stay before and after pneumonia index day were the secondary outcomes. We evaluated nephrotoxicity based on the new need and implementation of dialysis, including hemodialysis (HD) or continuous venovenous hemodialysis (CVVHD) during hospitalization after the pneumonia index date. The analysis of the length of ICU and hospital stays, and length of stay before and after pneumonia index day did not include patients who expired during hospitalization.

### Statistical analysis

Continuous variables were analyzed with the Mann–Whitney U test, and categorical variables were analyzed with chi-square test, or Fisher’s exact test. Kaplan–Meier analysis and log-rank tests were applied for comparison of survival between the CRAB and CSO AB groups. Statistical analyses were performed with SPSS software version 18.0 (SPSS Inc., Chicago, IL). A P value ≤ 0.5 was considered statistically significant.

### Ethics approval and consent to participate

The present study was approved by the Institutional Review Board (IRB) of the five participating hospitals, including IRB of Taipei Veterans General Hospital (2018-03-001CC), IRB of Tri-Service General Hospital (1-107-05-054), IRB of Kaohsiung Medical University Hospital (KMUHIRB-E(I)-20180141), IRB of China Medical University Hospital (CMUH107-REC3-052) and IRB of Taichung Veterans General Hospital (IRB CE18100A). The need of informed consent was waived by the Institutional Review Boards for the respective essence of this study, and the ethical principles and issues were in accordance with the Declaration of Helsinki.

## Results

### Prevalence of CSO AB and comparison of demographic characteristics

There were 955 patients with CR-GNB associated pneumonia enrolled initially in this study (Fig. 1). 380 patients were excluded according to the exclusion criteria including diagnosis of CAP/HCAP (64 cases), without ICU admission (156 cases), lung cancer with obstructive pneumonitis (8 cases) and non-CRAB pathogens (152 cases). Finally, 575 patients who were admitted to the ICU with CRAB-associated nosocomial pneumonia remained for following analysis. Among them, 79 cases were classified with CSO AB as the causative pathogen of pneumonia. Thus, the prevalence of CSO AB in CRAB-associated pneumonia in ICU patients is calculated to be 13.74% (79/575).

In Table 1, we compared the demographic characteristics of patients with CRAB and CSO AB pneumonias. Patients with CSO AB pneumonia were significantly more likely to receive intravenous (49.4% vs 29.0%, p = 0.001) or inhaled (50.6% vs 36.3%, p = 0.021) colistin therapy than patients with CRAB pneumonia. However, there was no significant difference between the groups regarding age, sex, body mass index, smoking, alcohol consumption, pneumonia types, ICU type, or comorbidities.

**Table 1:** Demographic characteristics of patients with CRAB and CSO AB pneumonia.

	CRAB (n = 496)	CSO AB (n = 79)	P value
Age, M (SD)	71.68 (14.70)	71.28 (16.37)	0.825
**Sex, n (%)**			
Female	170 (34.3)	30 (38.0)	0.607
Male	326 (65.7)	49 (62.0)	
Height, M (SD)	161.83 (11.69)	161.71 (9.52)	0.938
Weigh, M (SD)	61.16 (14.66)	59.89 (14.10)	0.497
BMI, M (SD)	23.25 (4.91)	22.85 (4.82)	0.518
Smoking	190 (38.6)	29 (37.2)	0.907
Alcohol consumption	98 (20.3)	13 (16.7)	0.548
**Pneumonia types, n (%)**			
HAP	136 (27.4)	21 (26.2)	0.985
VAP	360 (72.6)	58 (73.4)	
**ICU types, n (%)**			
Medical ICU	337 (67.9)	61 (77.2)	0.127
Surgical ICU	159 (32.1)	18 (22.8)	
**Comorbidities, n (%)**			
Malignancy	70 (14.1)	10 (12.7)	0.863
Heart failure	56 (11.3)	8 (10.1)	0.910
Renal insufficiency	95 (19.2)	12 (15.2)	0.493
Lung disease	85 (17.1)	15 (19.0)	0.808
Diabetes	172 (34.7)	34 (43.0)	0.189
Autoimmune disease	24 (4.8)	1 (1.3)	0.232
**Antimicrobial susceptibility**			
Colistin-sensitive	481 (99.4)	79 (100.0)	1.000
Tigecycline-sensitive	366 (75.9)	0 (0)	** < 0.001**
Ampicillin/sulbactamSulbactam-sensitive	212 (57.3)	0 (0)	** < 0.001**
Amikacin-sensitive	49 (19.4)	0 (0)	0.003
Susceptible to only 1 or 2 antimicrobial classes	156 (31.5)	79 (100.0)	** < 0.001**
**Antibiotics prescribed, n (%)**			
Intravenous colistin	144 (29.0)	39 (49.4)	**0.001**
Inhaled colistin	180 (36.3)	40 (50.6)	**0.021**
Carbapenem	205 (41.3)	31 (39.2)	0.820
Tigecycline	140 (28.2)	19 (24.1)	0.525
Ampicillin/sulbactamSulbactam	111 (22.4)	16 (20.3)	0.782
Amikacin	7 (1.4)	1 (1.3)	1.000

*M (SD)* mean (standard deviation).

Significant values are given in bold.

### Disease severities at initial presentation

In Table 2, we compared the disease severities, including APACHE II score, SOFA score, septic shock, mechanical ventilation, P/F ratio, dialysis and laboratory studies, and there was no significant difference between CRAB and CSO AB-associated pneumonia. Among these, APACHE II score was recorded at ICU admission; other variables were recorded on the pneumonia index date.

**Table 2:** Initial presentation of disease severity in patients with CRAB and COS AB pneumonia.

	CRAB (n = 496)	CSO AB (n = 79)	P value
**Disease severity**			
APACHE II score, M (SD)	22.42 (7.67)	22.69 (7.80)	0.782
SOFA score, M (SD)	7.80 (3.66)	7.39 (3.79)	0.357
**Presenting manifestations**			
Septic shock, n (%)	68 (13.7)	10 (12.7)	0.939
PF ratio, M (SD)	274.86 (131.58)	272.24 (125.45)	0.882
Mechanical ventilation, n (%)	429 (86.5)	67 (85.9)	1.000
Dialysis (HD + CVVHD), n (%)	96 (19.4)	17 (21.5)	0.766
**Laboratory studies, M (SD)**			
Leukocyte (× 10^9^/L)	12.9 (7.71)	14.5 (8.45)	0.084
C-reactive protein (mg/dL)	10.49 (8.10)	13.79 (26.72)	0.318
Albumin (g/dL)	2.73 (0.56)	2.73 (0.53)	0.953
Creatinine (mg/dL)	1.98 (1.96)	2.07 (1.96)	0.715

APACHE II score was recorded at ICU admission; other variables were recorded on the pneumonia index date.

*M (SD)* mean (standard deviation), *HD* hemodialysis, *CVVHD* continuous venovenous hemodialysis.

### Treatment outcomes

**Figure 2:**
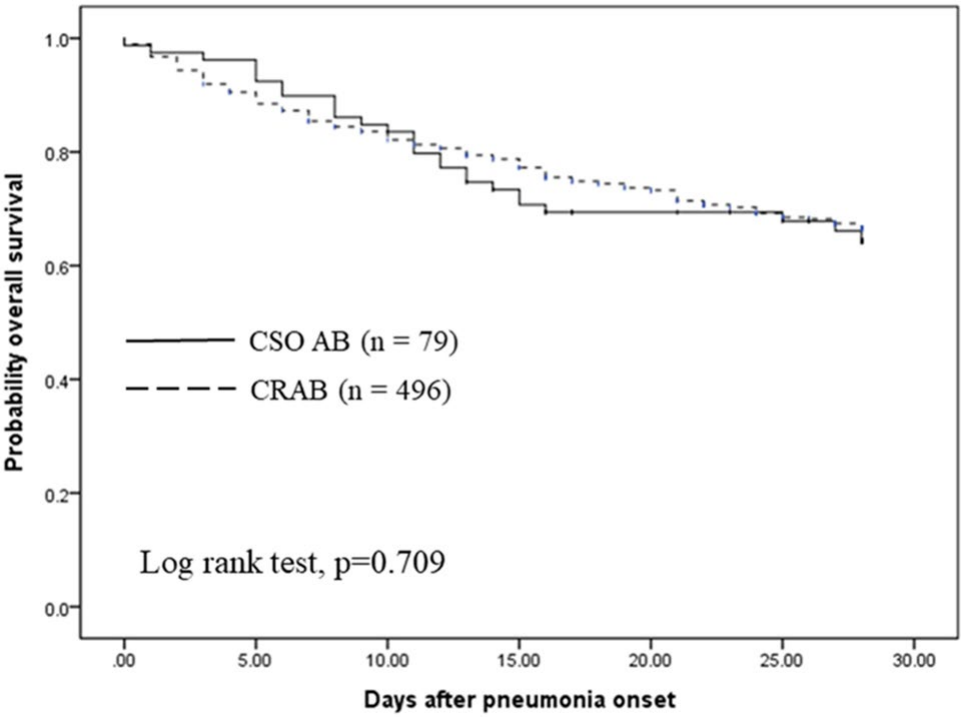
Kaplan–Meier analysis of 28-day survival in patients with CRAB and CSO AB pneumonia.

In Table 3, we compared the treatment outcomes between both groups. The in-hospital mortality was similar between CRAB and CSO pneumonia (46.4% vs 45.6%, p = 0.991). With regard to mortality at day 7, 14 and 28, it was similar between both groups. The CSO AB group had a significantly higher ratio of favorable clinical outcomes at day 7 than the CRAB group (65.8% vs 52.4%, p = 0.036), but this difference was not observed at day 14 and 28. The length of ICU stay was significantly longer in the CSO AB group than the CRAB group (27 days vs 19 days, p = 0.043), while the length of hospital stay was similar between groups. Furthermore, there was no significant difference regarding ventilator weaning at day 28, new need for dialysis, or microbiological eradication at day 7, 14, and 28, and the length of stay before and after pneumonia index day. In Fig. 2, the Kaplan–Meier analysis of 28-day survival between both groups shows non-significant difference (log rank test = 0.709).

**Table 3:** Treatment outcomes of patients with CRAB and COS -AB pneumonia.

	CRAB (n = 496)	CSO AB (n = 79)	P value
**Mortality (since pneumonia onset)**			
Day 7, n (%)	72 (14.5)	8 (10.1)	0.383
Day 14, n (%)	104 (21.0)	21 (26.6)	0.329
Day 28, n (%)	156 (31.5)	27 (34.2)	0.724
In-hospital mortality, n (%)	230 (46.4)	36 (45.6)	0.991
**Favorable clinical outcomes**			
Day 7	260 (52.4)	52 (65.8)	**0.036**
Day 14	260 (52.4)	43 (54.4)	0.833
Day 28	257(51.8)	37 (46.8)	0.483
**Microbiological eradication**			
Day 7	49 (18.0)	6 (13.0)	0.539
Day 14	131 (40.3)	22 (36.7)	0.700
Day 28	172 (50.0)	28 (47.5)	0.826
28-day ventilator weaning, n (%)	232 (46.8)	40(50.6)	0.375
Newly onset dialysis, n (%)	46(9.3)	4(5.1)	0.308
Length of ICU stay (days), M (R)	19(1–163) (n = 266)	27(6–80) (n = 43)	**0.043**^a^
Length of hospital stay (days), M (R)	51 (8–284) (n = 266)	55 (14–132) (n = 43)	0.667^a^
Length of stay before pneumonia index day (days), M (R)	9 (0–121) (n = 266)	8 (0–71) (n = 43)	0.894^a^
Length of stay after pneumonia index day (days), M (R)	35 (3–196) (n = 266)	41 (13–86) (n = 43)	0.597^a^

The analysis of the length of hospital and ICUICU and hospital stays, and length of stay before and after pneumonia index day did not include patients who expired during hospitalization.

*M (R)* median (range).

Significant values are given in bold.

^a^Mann-Whitney U test.

## Discussion

In 2007, Rios etc. observed that prior antimicrobial therapy and VAP are risk factors for acquiring CSO AB-associated VAP^12^. However, limited studies discussing the prevalence, presentation, disease severity, or outcome of CSO AB pneumonia were published after Rios’s report. This multicenter retrospective cohort study observed 13.74% of CRAB pneumonia were colistin-susceptible-only and patients with CSO AB and CRAB pneumonia had similar demographic characteristics and disease severities at initial presentation. Although patients with CSO AB pneumonia had significantly better clinical outcomes at day 7 and longer length of ICU stay than those with CRAB pneumonia, other treatment outcomes were similar, including clinical outcomes at day 14 and 28, mortality, microbiological eradication, ventilator weaning, and new need for dialysis.

*A. baumannii* is an concerning pathogen that has a staggering tendency for acquiring antimicrobial resistance to various categories of antibiotics by multiple mechanisms^6,17,18^. For CRAB in particular, Acinetobacter baumannii acquires carbapenem resistance by synthesis of carbapenemases, alterations in penicillin-binding proteins, loss of outer membrane porins, and overexpression of efflux pumps, among other mechanisms^19,20^. The *A. baumannii* resistance to carbapenems has been reported to be increasing during the last decade; more than 50 percent of Acinetobacter baumannii specimens isolated were carbapenem-resistant in most of the country^21–23^; the frequency of CRAB even reached 80–100 percent in south and southeast Asia^24^. The study regarding the epidemiology of CSO AB pneumonia, a subgroup of CRAB pneumonia, is scarce. Jacob etc. reported *Acinetobacter baumannii* accounted for 61% of CSO related infections, followed by *Klebsiella pneumonia* (24.4%), *Pseudomonas aeruginosa* (12.2%), and *Escherichia coli* (2.4%) in critically ill patients who were intubated and mechanically ventilated^25^. Our study further investigated the ratio of colistin-susceptible-only *A. baumannii* in CRAB pneumonia and observed that 13.74% (79/575) of CRAB were only susceptible to colistin. If clinicians could know the portion or percentage of colistin, tigecycline, or sulbactam-susceptible-only *A. baumannii* in CRAB infections in their hospitals or countries, appropriate empiric antimicrobial agents could be prescribed early when CRAB infections were suspected, a scenario that can possibly make the difference between mortality and survival in a critical care setting. Furthermore, pandrug-resistance (PDR) *A. baumannii* is another issue worthy to be concerned. PDR *A. baumannii* has been increasingly reported worldwide^26^, which could be, at least in part, associated with the high prevalence (33%) of colistin heteroresistance^27^ and the emergence of colistin resistance during treatment for *A. baumannii* infection^28^. The treatment options for PDR *A. baumannii* are limited and undetermined, that were primarily relied on the synergistic combination of antimicrobial agents^29–31^. High mortality (in hospital mortality rate: 79%) urges studies in future to investigate the effectively therapeutic regimen for PDR *A. baumannii* infection^32^.

Early prediction of CSO AB infection may help achieve better clinical outcomes by allowing early prescription of colistin. The delay of four days or more for colistin initiation lead to increase mortality in patients with VAP caused by CSO AB^33^. A prior episode of VAP and previous antimicrobial therapy more than 10 days have been reported as independent risk factors for acquiring CSO AB pneumonia^12^. However, these are also the risk factors for pneumonia caused by other multidrug-resistant pathogens, including CRAB^34–36^; other predictive factors, specific for developing CSO AB pneumonia, need to be identified and reported for clinical use. After comparing many variables representing demographic characteristics (Table 1) and disease severity (Table 2) between CRAB and CSO AB pneumonias, we detected no significant differences at initial presentation. It is worthy to investigate other predictive factors for developing CSO AB pneumonia by large randomized controlled studies in the future.

The in-hospital mortality of CRAB pneumonia in the present study was 46.4% (230/496), which is consistent with Zheng’s report (45.6%)^36^. We further explored the mortality of CSO AB pneumonia at days 7, 14, and 28, as well as in-hospital mortality, which were similar to that of CRAB pneumonia. A case–control study conducted by Samrah reported the 30-day mortality rate was 46.4% in patients with CSO AB associated VAP^33^, which was higher than the 28-day mortality rate (34.2) and close to the in-hospital mortality (45.6) of the present study. This disparity could be attributed to different characteristics of enrolled patients, for approximately one fourth were HAP patients in our study, while all patients were VAP in Samrah’s study.

We also observed that patients with CSO AB pneumonia had significantly better clinical outcomes at day 7 compared to patients with CRAB pneumonia. We hypothesize that significantly higher prescription of intravenous or inhaled colistin in the CSO AB group could attribute to this result, based on the evidence that colistin-based therapy may be associated with lower treatment failure rates^37^. However, the effect of better clinical outcomes at day 7 could not affect the longer ICU stay in patients with CSO AB pneumonia (Table 3), while longer stay in ICU of CSO infection was also reported in Jacob’s study^25^. We supposed patients with CSO infection may need longer period of intensive care compared to patients with non-CSO infection, but the precise causality needs to be further investigated. Furthermore, although more patients in CSO AB group received intravenous colistin therapy, there was no difference in new need for dialysis between both groups, which may be related to lower treatment failure at day 7 in the CSO AB group (less sepsis-induced kidney injury) or the occurrence of acute kidney injury but without the necessity for hemodialysis.

This is a multicenter study that minimized selection bias by taking different clinical settings and practices into consideration. However, some limitations exist. First, we calculated the prevalence of CSO AB pneumonia (13.74%) by the ratio of CSO to CRAB instead of *A. baumannii*, and that is analyzed according to the local pathogen data of Taiwan. Thus, the finding should be carefully extrapolated or applied for the concern of different microbial spectrum in different countries. Second, we did not collect prior antimicrobial therapy and previous VAP episode for baseline variable analysis, because these were reported as independent risks for developing CSO AB pneumonia in prior study^12^. Third, there were no detailed medical records regarding the APACHE II score at the pneumonia index date (only the date at ICU admission), so we could not compare the difference of APACHE II score at the time closest to pneumonia development. However, we analyzed the SOFA score at the pneumonia index date, which is also a good surrogate for disease severity. Fourth, we evaluated renal function impairment in both groups by new need for dialysis instead of acute kidney injury, which may underestimate the incidence of nephrotoxicity due to intravenous colistin, which was prescribed more frequently in the CSO AB group. Fifth, this study only recruited patients with nosocomial pneumonia caused by *Acinetobacter baumannii*, so the findings cannot be extrapolated to other pathogens. Sixth, polymicrobial infection and co-infection at other site are important issues to differentiate colonization versus true pathogen, and monomicrobial versus polymicrobial infection. However, we did not collect the associated variables for these differentiation. Seventh, the method for antimicrobial susceptibility to colistin was the BD Phoenix™ system or the VITEK® 2 system, instead of broth microdilution, a currently recommended method for colistin susceptibility. Eighth, we did not further apply multivariate analysis for the absence of survival differences in univariate analysis. Ninth, we did not further delineate the different antimicrobial therapeutic regimens in this study. However, we tried to simplify and keep the most essence/representation of regimen by illustrating as Table 1 (Antibiotics prescribed). Finally, due to an observational and retrospective study design, the bias regarding preference of clinicians for prescription of antimicrobial agents existed, such as colistin underused in the CRAB group.

## Conclusions

We provided more information regarding CSO AB pneumonia by observing that CSO AB accounted for 13.74% of all CRAB pneumonias, and the clinical presentation, disease severities and treatment outcomes were similar between CSO AB and CRAB pneumonia. Our findings will provide useful information for physicians when facing CSO AB pneumonia in clinical practice.

## Data Availability

The datasets used and/or analyzed during the current study are available from the corresponding author on reasonable request.

## Abbreviations

*A. baumannii*: *Acinetobacter baumannii*
APACHE: Acute physiology and chronic health evaluation
CRAB: Carbapenem-resistant *Acinetobacter baumannii*
CR-GNB: Carbapenem-resistant Gram-negative bacterial
CSO AB: Colistin susceptible-only *Acinetobacter baumannii*
CVVHD: Continuous venovenous hemodialysis
HD: Hemodialysis
ICU: Intensive care unit
VAP: Ventilator-associated pneumonia
WHO: World Health Organization
